# The Archives of Gynecology

**Published:** 1888-12

**Authors:** 


The Archives of Gynecology.—Seven hundred and twenty-eight is the record in numbers of the articles printed during 1888 in this valuable journal on the special subjects of its title. It is the aim of the editors to publish all current thought in the departments of Obstetrics, Gynecology, and Pediatrics. The publishers, Messrs. Leonard & Co., 141 Broadway, New York, do not send specimen copies, but if a subscriber is not pleased with the first number, it may be returned, and the order will be canceled. Subscription, $3.00 per annum. Payment is not asked till the end of the year.

